# How *Shift Work* Affects Our *Gut Microbiota*: Impact on *Gastrointestinal Diseases*

**DOI:** 10.3390/medicina61060995

**Published:** 2025-05-27

**Authors:** Angela Saviano, Marcello Candelli, Mattia Brigida, Carmine Petruzziello, Pietro Tilli, Francesco Franceschi, Veronica Ojetti

**Affiliations:** 1 Fondazione Policlinico Gemelli, IRCCS, 00168 Rome, Italy; marcello.candelli@policlinicogemelli.it (M.C.);; 2 Fondazione Policlinico, Tor Vergata, 00133 Rome, Italy; 3 Ospedale San Carlo di Nancy, GVM Research, 00165 Rome, Italy; carminepetruzziello@live.it; 4 Department of Internal Medicine, UniCamillus International Medical University of Rome, 00131 Rome, Italy; veronica.ojetti@unicamillus.org

**Keywords:** shift work, gut microbiota, dysbiosis, circadian rhythm, metabolic disease, occupational risk

## Abstract

*Background and Objectives*: Shift work and night work are common among emergency physicians. It is necessary to provide continuous care to patients, especially with acute diseases, including throughout the night. Literature studies show that shift and night workers have an altered light exposure, timing of sleep and intake of food. The consequence of this desynchronization with the biological clock can lead these workers to be more exposed to developing some acute and chronic health conditions. In particular, the alteration of the sleep–wake cycle, fatigue, the shortened sleep duration and the misalignment of the body’s hormone production is a codified risk factor of gut dysbiosis that can lead to acute and chronic diseases, also gastrointestinal ones. the aim of this narrative review is to collect and summarize evidence about the association between the disruption of the circadian rhythm, sleep and food timing alterations, gut dysbiosis and the risk of gastrointestinal diseases among shift and night workers. *Materials and Methods*: we searched for evidence about the association of shift and night work, dysbiosis, gut microbiota and gastrointestinal diseases among shift workers in healthcare settings. *Results*: shift work and night work are associated with a higher risk of diseases, an inflammatory state and the alteration of the gut microbiota composition; but definitive data are still inconsistent. *Conclusions*: Until now, obtaining conclusive results in regard to the relationship between shift work, the gut microbiota and the increased risk of gastrointestinal disorders has been particularly complex and not yet feasible. More confirmatory studies are needed to better characterize risk factors and realize preventive measures.

## 1. Introduction

Shift work and night work are common in the emergency department. Shift work denotes a job-plan in which physicians and other staff members work in hours that differ from the normal working schedule of 9 a.m. to 5 p.m.; it usually comprises rotating shifts, on-call or casual shifts, regular evening or night schedules, split shifts, irregular schedules, 24 h shifts and other no-day work schedules [1]. In particular, night work has been defined as work that covers at least 3 h between 11 p.m. and 6 a.m. [2]. The literature has reported that from 15 to 30% of workers carry out a shift work schedule [3], with about 20% of night shift work for a total of 0.7 billion globally [4]. Shift work inevitably disrupts the circadian rhythm (approximately 24 h) that is the normal sleep–wake cycle, with consequently less time for sleep, more fatigue and risks of adverse health effects (i.e., gain weight, metabolic syndrome, type 2 diabetes [5], cardiovascular diseases [6], mood disorders [7] and cancers). In addition, a very interesting point is that shift workers are also at a greater risk of gastrointestinal diseases, injuries during work and problems with their social and familial life [8]. Recent findings have shown that alterations of circadian rhythms are associated with altered intestinal gut microbial communities (i.e., dysbiosis) with subsequent inflammatory states, metabolic disfunction and a risk of gastrointestinal disorders [9]. A study by Knutsson A. et al. [10] reported an association between one-year shift work and gastrointestinal disorders and peptic ulcers. Few studies have analyzed the association with gastroesophageal reflux disease, chronic inflammatory bowel diseases or gastrointestinal cancers. And, even if literature reported that shift workers experienced a higher prevalence of gastrointestinal symptoms (questionaries with increased scores for abdominal pain, diarrhea or constipation, sense of nausea and regurgitation) with an overall picture that seemed to indicate a significant association, it is essential to consider that these studies are few and insufficient in terms of their control groups and are not standardized in their collecting of samples, study design and diagnostic methods. The aim of this narrative review is to collect and summarize evidence about the association between the disruption of the circadian rhythm, sleep and food timing alterations, gut dysbiosis and the risk of gastrointestinal diseases among shift and night workers.

## 2. Methods

This narrative review included studies published in any language over the last 10 years on the topic of shift work, the circadian rhythm, gut microbiota and diseases, mainly focusing on gastrointestinal diseases. We searched clinical trials, systematic reviews and observational studies (case–control, case series, longitudinal and cross-sectional studies). We conducted our research considering the title, abstract, type of study and period of research. We searched on PubMed^®^, Web of Science^®^, Up-to-Date^®^ and Cochrane^®^. No ethical approval was required to carry out this review. The main words we searched for were as follows: shift work AND gastrointestinal diseases; gut microbiota AND circadian rhythm; shift work AND circadian rhythm AND/OR gut microbiota; gut clock AND gastrointestinal diseases; emergency department AND night shift work AND/OR gut microbiota AND metabolism; night shift work AND nutrition AND dysbiosis; and shift work AND emergency department AND gastrointestinal diseases.

### 2.1. Role of Gut Microbiota and Circadian Rhythms

The gut microbiota balance could be influenced by dietary patterns, drugs, stressors, sleep habits and light exposure [11]. Studies underline that the light/dark cycle is essential for the correct functioning of the central clock (located in the brain in the hypothalamic suprachiasmatic nucleus) and for the subsequent correct functioning of the peripheral clocks (in the rest of body, including intestines) which receive signals [7]. Gut bacteria have shown diurnal fluctuations in regard to their abundance and functions. Poor sleep habits could affect the composition of the gut microbiota and the production of metabolites, butyrate, vitamins with subsequent poor digestion, gastric activities and alterations in the absorption of nutrients. Some genes have been studied as “clock genes”, existing in almost every cell. They are the circadian locomotor output cycles kaput gene (clock), genes period 1/2/3 (Per1/2/3), cryptochrome 1/2 (Cry1/2) and brain and muscle aryl hydrocarbon receptor nuclear translocator-like 1 (Bmal1) [8]. The clock genes, crucial for the coordination of gut rhythmic functions, are well represented in the gastrointestinal tract [9]. The gut “clock” cells are mainly entrained by the time of feeding more than the clock in the suprachiasmatic nucleus [12]. As a consequence, the alteration of eating times (common among shift workers) could lead to the desynchronization among the circadian biological clocks: this mechanism has been hypothesized as responsible for the gastrointestinal diseases among shift workers. The literature shows that the alteration of the circadian rhythm, sleep loss, sleep fragmentation and total sleep deprivation represent a physiological “stressor” due to changes in the hypothalamus–pituitary–adrenal (HPA) axis and cortisol levels [13]. Experimental studies conducted on mice identify chronic sleep alterations and circadian misalignments as responsible for gut microbiota changes, with a subsequent cascade of low-grade inflammation, increased gut permeability and systemic bacterial migration. For example, rats subjected to sleep deprivation for three–five weeks developed more tissue infections and a greater invasion of bacterial species in the mesenteric lymph nodes [14]. The latter was associated with the alteration of gut microbiota communities with the translocation of bacteria and an invasion within the first five days of sleep deprivation [15]. Other studies conducted in animal models confirmed an increased gut microbiota dysbiosis in cases of a loss of the circadian rhythm [16]. In particular, a high number of Firmicutes has been detected in cases of insufficient sleep. In humans, there is a lack of studies regarding the link between sleep loss, gut dysbiosis and subsequent gastrointestinal diseases.

### 2.2. Circadian Rhythm Dysregulation and the Effect of Melatonin on the Gut

Melatonin is a hormone primarily synthesized in the pineal gland. Melatonin interacts with the suprachiasmatic nucleus of the hypothalamus and the retina, regulating the body’s sleep–wake cycles [7]. Studies underline that melatonin has been associated not only with sleep promotion but also with the regulation of gastrointestinal motility, with a potential role in gastrointestinal diseases [12]. An interesting study conducted by Song et al. [17] on a murine model utilized an intraperitoneal administration of melatonin and showed a significant inhibition of stress-induced defecation. The authors suggested that this inhibitory effect could originate from an antagonistic effect of melatonin towards the stress-induced release of serotonin and corticotropin-releasing factor secretion [17]. Similar evidence comes from a randomized, double-blind, placebo-controlled study on human subjects, by the same authors [18]. In this study protocol, the effects of melatonin in treating abdominal symptoms and sleep alteration were assessed in 40 patients with inflammatory bowel syndrome (IBS) and sleep disturbance. Patients were randomized 1:1 to receive 3 mg of melatonin daily for 14 days at bedtime or a placebo. Patients registered a questionnaire (bowel symptoms, quality of sleep and the psychological status) with rectal manometry and overnight polysomnography. After 2 weeks, patients receiving the treatment with melatonin had a significantly greater decrease in abdominal pain and significantly improved their sense of urgency and pain, compared with patients receiving the placebo (no differences between the two groups in the quality and quantity of sleep). In addition, there were no differences in the sensation of anxiety and depression between the two groups. The authors of the study conclude that melatonin is effective in reducing abdominal symptoms and rectal pain in patients affected by IBS, without significant effects on their sleep and psychological status. Based on a randomized, double-blind, placebo-controlled study [18], melatonin improves abdominal pain in irritable bowel syndrome patients who have sleep disturbances.

### 2.3. Circadian Rhythm Alteration, Hormone Imbalance and Gastrointestinal Diseases

The circadian system is fundamental for the coordination of satiety and the metabolism of nutrients, including processes in which gut hormones are involved. The circadian system orchestrates daily rhythms in gastrointestinal processes, including digestion, absorption, motility, hormone secretion, barrier functions and the gut microbiota. There is a scarcity of human studies that separate the effects of behavioral cycles, such as feeding–fasting or activity–sleep patterns, from those of the intrinsic circadian clock. The feeding time serves as the most crucial cue for aligning peripheral clocks, with gut hormones—especially insulin and IGF1—playing a pivotal role in transmitting phase information to these clocks. Interventions like time-restricted eating, scheduled physical activity and the use of chrono-biotics—substances that modulate the phase, amplitude or period of the circadian system—show potential for addressing chronodisruption and related health issues. The circadian system governs 24 h rhythms in various molecular, physiological and behavioral processes. The central clock resides in the suprachiasmatic nucleus of the hypothalamus and is entrained by the light–dark cycle. This master clock coordinates peripheral clocks found in tissues and organs, with additional environmental factors—most notably feeding times—serving as key synchronizers for these peripheral clocks. This synchronization enables the body to anticipate predictable environmental changes, such as nutrient availability, during regular feeding periods. The current knowledge highlights the role of circadian clocks within the digestive tract and its accessory organs in regulating gastrointestinal processes. These include digestion, motility, hormone secretion, barrier functions and the composition of the gut microbiota. The intricate interaction between circadian clocks in the digestive system influences glucose homeostasis, lipid metabolism and bile acid regulation. Furthermore, there is a bidirectional relationship between the central clock and peripheral digestive clocks, which is mediated by various entraining factors. Behavioral strategies, such as adjusting feeding patterns, or pharmacological interventions, like chronobiotics, may offer promising approaches to mitigate the harmful effects of chronodisruption. Feeding, an essential process for all animals, is regulated by homeostatic mechanisms and is further influenced by the circadian (~24 h) cycle. The brain uses a network of circadian clocks to establish daily windows for food consumption, which generally align with the active phase of the organism [19]. Central to this regulation is the master clock in the suprachiasmatic nuclei of the hypothalamus, synchronized by the light–dark cycle, alongside secondary clocks located in the hypothalamus and brainstem. Rhythmic cues—such as metabolic hormones, circulating nutrients and visceral neural inputs—facilitate the communication between the brain and peripheral organs through intricate molecular interactions linking metabolic processes with the circadian system [20]. These dynamic interactions highlight the detrimental effects of chronodisruption and mistimed eating on metabolic health. Conversely, consuming food during the active phase, even if the diet is unbalanced, helps reduce metabolic disturbances. Thus, meal timing, in addition to energy intake and the dietary composition, is crucial to avoid circadian desynchronization and minimize metabolic risks [21]. This perspective emphasizes the dual role of homeostatic and circadian processes in regulating food intake, outlining mechanisms governing feeding times while underscoring the metabolic benefits of proper timing and the risks posed by mistimed eating [22].

### 2.4. Mechanisms by Which Shift Work Could Determine Gastrointestinal Disturbances

The gastrointestinal system is characterized by a circadian rhythm. This regards bowel movements, the secretion of juices (gastric bile…) and the synthesis of biliary acids and hormones [23]. Furthermore, the circadian clock can influence the regulation of appetites and satiety. Studies have shown that gastric motility has a peak at midday and its lowest level in the night (and gastric secretion was high in the evening and low in the morning; a bile secretion during the daytime at about 12 a.m. and 8 p.m. with a persistent peak in case of fasting) or colonic movements are increased during the day after meals [19]. The production of hormones has a circadian rhythm, too. For example, pepsinogen is released from the stomach and is transformed, through hydrochloric acid, in pepsin (an enzyme that digests proteins and that can cause peptic ulcers, acting as an aggressor). Gastrin is a hormone that promotes the secretion of hydrochloric acid (a gastric acid). A study conducted on shift workers (schedule: morning shift from 06:00 a.m. to 02:00 p.m., afternoon shift 02:00 p.m.–10:00 p.m. and night shift 10:00 p.m.–06:00 a.m.) compared to a control group (workers from 07:45 a.m.–04:45 p.m.) underlines increased blood levels of serum gastrin and pepsinogen compared to the control group [24]. Another study reported that the infection of *Helicobacter pylori* was more prevalent (46.1% vs. 34.6%) in shift workers than in day workers [25]. The prevalence of peptic ulcers in *Helicobacter pylori*-infected shift workers was higher (28.7% vs. 9.3%) than in infected day workers [26].

### 2.5. Dysbiosis and Gastrointestinal Diseases

It is well known that the condition of “dysbiosis”, which consists of an altered composition and function of the gut microbiota, is associated with many acute and chronic gastrointestinal diseases [27]. The open question is whether dysbiosis represents the cause or the effect of these diseases. The study of the gut microbiota and of some medical conditions, such as inflammatory bowel disease (IBD), diverticulitis, fatty liver disease and gastritis, related to *Helicobacter pylori* and celiac disease share the idea that the gut microbiota can be altered due to many factors, from diet to drugs and from toxins to pathogens, and also to a variety of environmental factors. Interesting studies on animal models colonized by enteric pathogens show that the alteration of the balance of the gut microbiota can lead to both local and systemic inflammation, triggering the breaking of the gut barrier, leaky gut and, sometimes, autoimmunity and metabolic conditions. In addition, the technology (culture, fecal samples and 16S rRNA) to classify gut bacteria, viruses and archaea show that each individual has their own profile of communities of gut microorganisms (the gut microbiota differs substantially among individuals). A metagenomic analysis revealed that a percentage of microbial genes (about 40%) are shared with almost half of the general population, representing the core microbiome. The remaining part differs among individuals, also due to genetic differences. Considering the phylum level, Firmicutes and Bacteroidetes are the most represented, then *Actinobacteria*, *Proteobacteria*, *Verrucomicrobia* etc. [15]. As stated above, may factors can lead to an imbalance of the gut microbiota composition, such as diet, drugs (antibiotics), infections, stress and sleep–wake cycle alterations, with the alteration of essential processes triggering some gastrointestinal diseases, such as Chron’s, ulcerative colitis and Clostridium Difficile infections [1]. In this context, the restoration of a healthy gut microbiome is essential. Dietary interventions, probiotics, prebiotics and symbiotics could represent a useful strategy for the management of gut-related diseases [28]. The development and the progression of various gastrointestinal diseases is influenced by the perturbations of the gut bacteria structures and their metabolic functions [3]. Gastrointestinal symptoms in patients with IBS recognize the same alteration of the gut microbiota composition and intestinal environment [2].

### 2.6. Food Intake and Circadian Rhythm: Chrononutrition

Studies underline that the meal timing is a crucial factor that influences metabolic health, due to the close interaction between our circadian clock and metabolic homeostasis [29]. The daily feeding time is defined as the period from the start of the first meal to the end of the last meal. It is known that feeding is regulated by homeostatic mechanisms, such as food consumption which is coordinated by the brain based on the circadian cycle of 24 h. Modern society [30] is characterized by a large availability of food and the continuous exposure to artificial light, and the continuous disruption of sleep is responsible for the alteration of the day–night biological rhythm with the subsequent development of cardiometabolic diseases (also, type 2 diabetes) [31]. The human metabolism functions better with an energy intake early in the morning, when the glucose tolerance and insulin sensitivity are higher. In adipose tissue and skeletal muscles the molecular clocks are regulated by nutrition and food intake [32]. Human cells, with the evolution and the changes in meal times and light exposure, developed systems of adaptation. Brain clock genes regulated the control of food intake through the suprachiasmatic nuclei of the hypothalamus and secondary clocks in brainstem regions in combination with light signals [5]. On the other hand, metabolic hormones, neural signals and circulating nutrients in the blood retransmit cues so that the brain and peripheral organs can be synchronized to the time of feeding. These interactions are complex and not completely understood. Timed meal patterns, together with the dietary composition and energy intake, are critical to prevent circadian desynchronization and limit metabolic risks [33]. Desynchronization leads to an increased risk for obesity, type 2 diabetes and cardiovascular diseases. From the gastrointestinal tract to the brain, signals are integrated and translated into chemicals molecules and neuropeptide releases. The secretion of hormones (corticosterone, insulin, glucagon, adiponectin, ghrelin, leptin…) is involved in the regulation of metabolic processes, such as the sense of satiation or an appetite [4]. These hormones show changes after postprandial meals, mirroring the pattern of the food intake. When these complex interactions are disrupted due to shift work, traveling abroad and changed meal timings, a desynchronization between the central and peripheral clocks can occur, leading to metabolic health side effects. The literature reveals that food intake is connected to the peripheral clocks of the liver, heart and pancreatic tissue [34]. The feeding time, under normal conditions, is aligned with the external light–dark cycle, contributing to the connection of peripheral oscillators with the central clock [35].

## 3. Results

**Figure 1:**
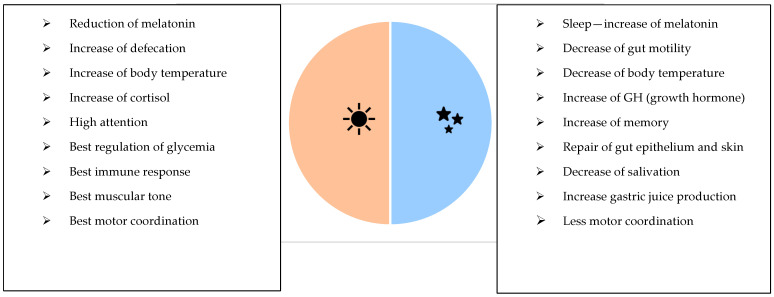
Circadian body rhythm.

**Figure 2:**
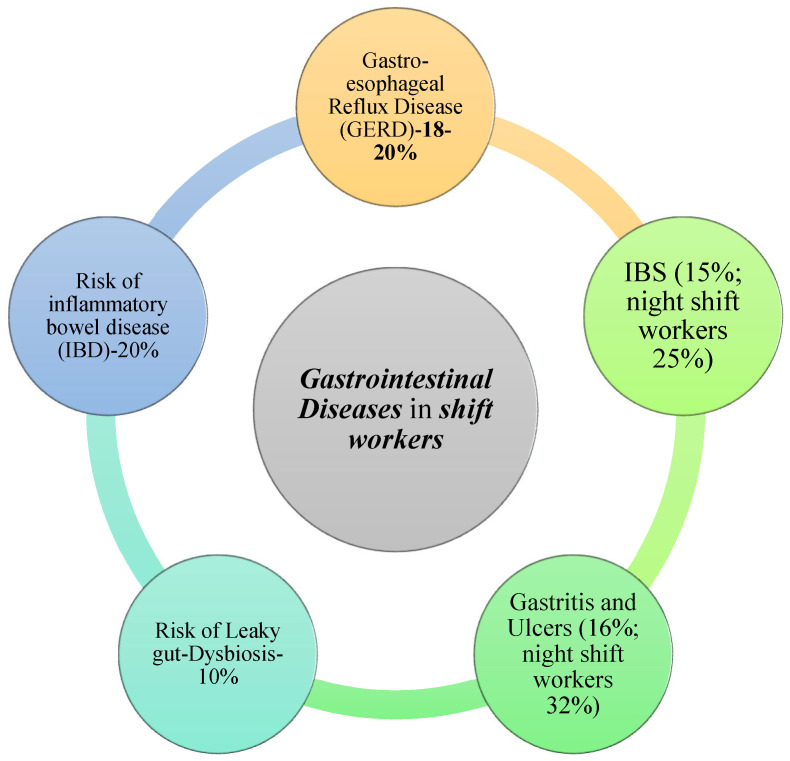
Most common gastrointestinal diseases and percentage (%) reported in shift workers [8,36,37,38,39].

This narrative review highlights some key points. The disruption of the circadian rhythm (Figure 1), linked to shift work and night shift work, negatively impacts the health of workers by exposing them to cardiovascular, metabolic and gastrointestinal diseases (Figure 2) and cancer. Sleep disturbances, an altered time and quality of meals, a poor diet and dysbiosis are only some of the most frequent etiological factors, associated with shift and night work, that require attention, regulation and correction. Preventive and management measures are therefore necessary to reduce health risks and improve workers’ short- and long-term outcomes.

## 4. Discussion

**Figure 3:**
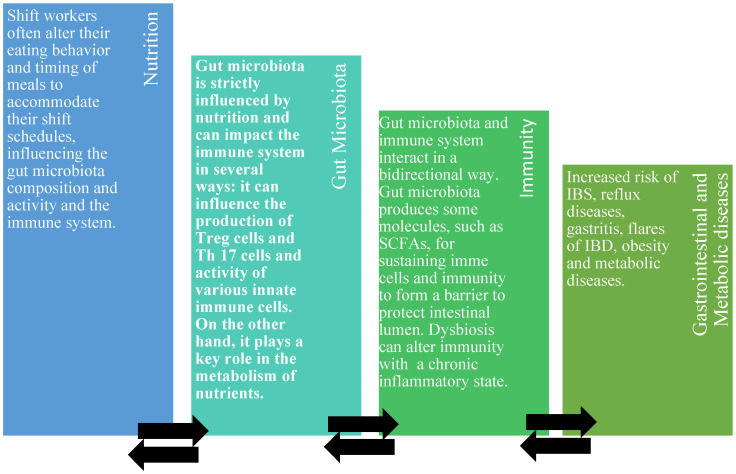
Nutrition, gut microbiota and immunity in shift workers.

Healthcare workers represent the largest proportion of shift workers to provide 24 h/day medical assistance [40]. Shift work and night shift work can have negative implications on health [27]. Night shift workers suffer the inversion or alteration of the sleep–wake cycle, with the subsequent stress of the endogenous regulation of the biological circadian rhythms. These functions are driven by the body’s central clock, located in the suprachiasmatic nucleus (SCN) of the hypothalamus [1]. The SCN can be considered as the principal circadian pacemaker in individuals, which is able to generate circadian rhythms during rest and activity times, as well influence the core body temperature, the neuroendocrine and autonomic systems, memory and psychomotor activities and other behavioral and physiological processes [31]. The SCN is controlled by environmental factors such as the “light–dark” cycle. The physiological condition consists of being awake during the day and sleeping during the night [30]. Night shift workers must adapt to the new rhythms, with notable changes in biological functions [8]. Functions normally active during the daytime and put on pause at night are reversed, requiring workers to undergo an adjustment [41]. However, the rate of adjustment can vary significantly based on the duration and schedule of the night shifts. While workers on rotating shifts must frequently adapt to varying periods, those committed solely to night shifts can adjust more effectively by consistently maintaining an inverted sleep–wake cycle [42]. The misalignment of the circadian rhythm due to shift work and night shifts often results in sleepiness, fatigue, insomnia, digestive problems, impaired cognitive performance, irritability and diminished efficiency. Although shift work does not alter the overall caloric intake, it does affect eating patterns, including meal frequency and timing, with a negative metabolic impact on health [43]. Additionally, sleep deprivation may lead night shift workers to consume higher-fat and carbohydrate-rich foods, coupled with increased snacking during short breaks. After problems related to sleep, digestive issues affect 20–75% of night shift workers, compared to only 10–25% of day workers [44]. These problems result from a misalignment between mealtimes and gastrointestinal functions regulated by the circadian rhythm, with subsequent alterations in the microbiota composition, dysbiosis and leaky gut [11]. Some of these functions include the secretion of gastric, bile and pancreatic fluids; intestinal movement; enzyme activity; nutrient absorption; and the release of hunger-related hormones [19]. Furthermore, night shift workers skip normal meals (breakfast, lunch and dinner) preferring snacking, which is more practical during the night. They often consume lower-quality foods (highly processed snack foods, sugar-sweetened beverages, high glycemic foods and fried foods), frequently opting for pre-packaged or highly processed items with a higher preservative content. They also experience gastrointestinal issues more frequently than their daytime counterparts due to these habits [45]. These gastrointestinal disorders range from mild symptoms, such as altered bowel habits, indigestion, flatulence and heartburn, to more severe conditions like peptic ulcers, irritable bowel syndrome (IBS) and gastritis. Nutrition represents a link between the gut microbiota and the health of shift workers [46]. Diets set the composition of the human gut microbiota, which in response produces active metabolites able to regulate gastrointestinal homeostasis and the immune system (Figure 3). This latter can protect or prevent health or disease or, in the condition of dysbiosis, lead to a chronic inflammatory state [46]. The diet of shift workers should pay attention to the quality of food, the time of meals and meal frequency and regularity. In order to accommodate shift schedules, shift workers alter their eating behavior with poor and irregular dietary patterns. Moreover, they consume food during the night, with an increased risk of obesity and metabolic syndrome. The habits of eating during the night have a negative effect on the circadian rhythm and induce changes in the metabolism and changes in the gut microbiota composition [46].

Additionally, the challenges of night shift work, including disrupted social patterns and psychological stress, often intensify these negative effects. Factors like stress and interpersonal conflicts further compound the impact not only on gastrointestinal health but also on metabolic and cardiovascular health, making night shift workers particularly vulnerable to these disturbances. In fact, disrupted sleep patterns can lead to metabolic disorders, hypertension, cardiovascular diseases and diabetes type 2. It is estimated that night shift workers have, on average, a 40% higher chance of developing ischemic heart disease compared to day workers [47]. Moreover, shift workers reduce their physical activities with a subsequent unhealthy status. Furthermore, circadian rhythm disruptions, poor sleep quality, digestive problems and altered lifestyle habits, such as irregular meal timing, the lower nutritional quality of food and increased snacking, can lead to weight gain, elevated triglycerides and higher cholesterol levels. Additionally, lifestyle factors are involved, such as smoking, obesity and high cholesterol, which can exacerbate cardiovascular risks and cancer. In 2007, the International Agency for Research on Cancer classified night work with circadian disruption as a probable human carcinogen [48]. In 2008, some Danish women who had worked the night shift, obtained the recognition of the occupational health for breast cancer. In addition, shift workers have a higher risk of prostate cancer and colon rectum cancer, too [48]. So, it is essential to adopt preventive measures against chronodisruption in order to protect shift workers from the risk of cardiovascular, metabolic and gastrointestinal diseases and cancer. The data on the exposure to night shifts often rely on sporadic self-reports or general associations with industries where shift work is common. Details such as the frequency of night shifts per month, consecutive night shifts and the duration of duty periods are typically absent from these analyses, requiring new studies to explore these issues. The above findings primarily come from a few cross-sectional epidemiological studies. However, these studies often lacked standardized diagnostic criteria and failed to account for confounding variables, like age, smoking habits and socio-economic status. Regardless of this, appropriate preventive interventions [49] in the organization of shift schedules are necessary to protect the health of shift workers. It is recommended to maintain, as much as possible, a consistent sleep schedule, even on days off. It is suggested to obtain 7–9 h of quality sleep before shift work. Another important issue should be to create a sleep-friendly environment: sleeping, for example, in a dark and quiet area, using black-out curtains and ear plugs to minimize disruption. Careful health surveillance and social and psychological support can be one of these corrective measures that allow workers to keep working without significant health impairments. In addition, useful mindfulness and stress management practices should be performed through relaxation techniques, such as yoga and meditation. Shift schedules should be tailored to specific personal characteristics, such as the age of workers, socio-economic status and background of the involved workers. In addition, together with check-up screening, additional rest breaks to allow meals and naps or holidays or supplementary rest days can help shift workers to improve their recovery, with beneficial effects on their physical and mental health. This field has acquired a growing importance in recent decades, but more research and studies are needed to improve, at several levels, environmental conditions, human homeostasis and shift workers’ well-being.

## 5. Conclusions

Shift work has been associated with a higher risk of diseases, an inflammatory state and alterations in the gut microbiota composition. It seems that digestive issues affect 20–75% of night shift workers, compared to only 10–25% of day workers. However, current studies exploring the relationship between shift work, the gut microbiota and increased risks of gastrointestinal disorders are still inconsistent, and, until now, providing conclusive results is particularly complex and not yet feasible. More confirmatory studies are needed to support this evidence to better characterize risk factors and realize preventive measures.

## Data Availability

No new data were created or analyzed in this study.
